# Supplementary material for: Implementing public health emergency operations centres according to an international framework in Ethiopia, Nigeria, and Senegal: Best practices and achievements, 2021
Source: PLOS Glob Public Health. 2026 Apr 27;6(4):e0006211. doi: 10.1371/journal.pgph.0006211 (PMC13119900; doi:10.1371/journal.pgph.0006211)
Supplement: S1 Text — (PDF) [file pgph.0006211.s001.pdf]

Welcome to the Institutional Survey

We are conducting this survey to document the development and implementation of your PHEOC, its role in responding to the COVID-19 pandemic, as well as to address the gaps and needs in PHEOCs best practices guidance, and to provide policy recommendations for implementing and strengthening PHEOC to promote public health emergency preparedness and response in Africa region.

This study is conducted by the World Health Organization.

There are two sets of questionnaires for this study, the institutional survey and the individual capacity survey.

This is the Institutional Survey. It is recommended that these questions be completed by the PHEOC manager, or designated staff supported by the PHEOC manager.

In the survey, you will be asked to review the implementation of your PHEOC, including policies, plans, organizational structure, human resource, information system, infrastructure, activities and its role in epidemic response, etc.

Some basic information about your personal background will be collected to track the progress of the survey, including your contact name, position, email address, etc.

After the survey, we would also like to kindly ask you to send us a digital copy of your PHEOC's operational documentations (organizational chart, policies, strategic plans and procedures, internal guidelines, handbooks, training materials, flyers and posters, IAPs, etc.) so we could have a more comprehensive understanding of your Center's daily operation.

You can download the PDF version of this survey from the link below:
https://bit.ly/33b0FX0

Thank you for participating in this survey, your feedback is very important.

General information of survey respondent

We collect these information to ease the tracking and analysis of the survey.
Only the research team will have access to the information you provide below.
We are trying our best to protect your privacy.

* In which country do you work?

- ◯ Ethiopia
- ◯ Nigeria
- ◯ Senegal

* In what province do you work?

[ ]

* What is the name of your institution?

[ ]

* Your contact name:

[ ]

* Telephone/Fax:

[ ]

* Email Address:

[ ]

[Part A] Institutional Information

- A1. Development of your PHEOC

The Part A of this survey asks for general information of the implementation and development of your PHEOC, including basic characteristics, inner and outer settings and basic human resource structures. Relevant personnel responsible for specific area of work should be involved in completing the survey.

* A1.1 Date of PHEOC establishment

Please specify the date below

Date

MM/DD/YYYY

* A1.2 Which government agency led the PHEOC establishment?

- [ ] Ministry of Health (Health Sector)
- [ ] Ministry of Disaster Management
- [ ] Ministry of Development and Reform
- [ ] National CDC
- [ ] Other (please specify)

* A1.3 Where did funding come from for the PHEOC establishment?

- [ ] National funds
- [ ] International aids
- [ ] Regional aids
- [ ] Donors
- [ ] Other (please specify)

* A1.4 How was the legal authority established?

- ○ By an executive directive (from a chief executive or minister) within the responsible agency
- ○ By a directive from the government or from the head of state
- ○ By legislation
- ○ By fiscal appropriation
- ○ Other (please specify)

* A1.5 Do you have a national standing policy group oversighting the PHEOC?

◯ Yes ◯ No

* A1.6 What sectors are involved in the standing policy group? Please list all of them below:

* A1.7 Does the standing policy group involve downstream emergency response units (tactical response units)?
If yes, what are the response units involved?

Do you have any other inputs or comments for the questions on this page?
(e.g if any answer exceeded the character limits, or if you have any other relevant comments, feel free to put them here.)

[Part A] Institutional Information
- A2. Characteristics of your PHEOC

* A2.1 Which is the managing level of your center?

- ◯ National-level
- ◯ Subnational-level
- ◯ Site-level
- ◯ Other (please specify)

* A2.2 Which of the following features does your center have? (Select all that apply)

- ☐ An annual process for review of national risks and resources
- ☐ A robust and ongoing training programme, ensuring that all core staff members function at an expert level
- ☐ 24/7 readiness for activation within 120 minutes
- ☐ Redundancy in personnel for all IMS positions, permitting sustained and continuous operations (24/7/365)
- ☐ Surge personnel from other work centres who are trained to support and sustain operations
- ☐ Able to support regional coordination
- ☐ Procedures for accessing extra-jurisdictional resources

* A2.3 What risk assessments have been conducted at your PHEOC?

- ☐ VRAM (Vulnerability and Risk Assessment and Mapping)
- ☐ STAR (Strategic Tool For Assessing Risk)
- ☐ THIRA (Treat Hazard Identification Risk Assessment)
- ☐ No risk assessment have been conducted
- ☐ Others, please specify all that are not mentioned above:

* A2.4 Which phase(s) does the PHEOC cover? Check all that apply.

- ☐ Prevention
- ☐ Preparedness
- ☐ Response
- ☐ Recovery
- ☐ None of above is covered.

* A2.5 Is the planning process at your PHEOC based on these risk assessments?

◯ Yes ◯ No

* A2.6 Please specify the key plans of your PHEOC:
(e.g. Emergency preparedness and response plan, hazard specific plan, PHEOC plan/PHEOC handbook, Standard Operation Procedures, communication plan, continuity of operations plan etc.)

Do you have any other inputs or comments for the questions on this page?
(e.g if any answer exceeded the character limits, or if you have any other relevant comments, feel free to put them here.)

[Part A] Institutional Information
- A3. Inner setting of your PHEOC

* A3.1 Which level of facility does your center have?

- ◯ As needed, convertible space or mobile.
- ◯ Dedicated facility. Core hours of operation 08:00–17:00.
- ◯ Dedicated facility. 24/7/365 operation.

* A3.2 Please specify the space available for EOC in square meters (m^2):

____

* A3.3 How many of the following space does your center have? (Input 0 for the spaces that your Center does not yet have.)

Operations room

____

Call center

____

Conference room

____

Meeting rooms

____

Equipment room

____

Storage areas

____

Rest, sanitary and food service areas

____

Space for watch staff

____

Space for media briefing (delegated or designated media briefing room)

____

* A3.4 Do(es) the **Operation room**(s) in your PHEOC have sufficient work stations?

◯ Yes ◯ No

* A3.5 Where does [funding for PHEOC daily operation] come from?

- [ ] National funds
- [ ] International aids
- [ ] Subnational funds
- [ ] Regional aids
- [ ] Other (please specify)

* A3.6 Do you have enough funds to sustain the PHEOC?

- ( ) Yes
- ( ) No

* A3.7 What is the annual budget for the PHEOC? (in US dollars $)

* A3.8 Did the Center's operation remain within annual budget?

- ( ) Yes
- ( ) No
- ( ) Do not know

* A3.9 If the Center's operation ran under budget, how were savings made?

* A3.10 If the Center's operation ran over budget, why did this happen? How did this resolve?

* A3.11 Is working environment conducive to effective productivity?

- ( ) Yes
- ( ) Somewhat
- ( ) No

* A3.12 How would you rate the teamwork and morale of your team?

- ( ) Very good
- ( ) Good
- ( ) Fair
- ( ) Poor
- ( ) Very poor

* A3.13 How would you rate the level of communication within your team?

- ( ) Very good
- ( ) Good
- ( ) Fair
- ( ) Poor
- ( ) Very poor

Do you have any other inputs or comments for the questions on this page?
(e.g if any answer exceeded the character limits, or if you have any other relevant comments, feel free to put them here.)

[Part A] Institutional Information

- A4. Outer setting of your PHEOC

* A4.1 Please specify the population size your center serves:

* A4.2 Which type of hazards have your center dealt with so far? (Select all that apply)

- [ ] Geophysical: Earthquake
- [ ] Geophysical: Tsunami
- [ ] Geophysical: Geophysical-trigger Mass movement (landslide, rock fall, subsidence, etc)
- [ ] Geophysical: Liquefaction
- [ ] Geophysical: Volcanic activity
- [ ] Hydro-Meteorological: Flood
- [ ] Hydro-Meteorological: Hydro-meteorological-trigger Mass movement (landslide, avalanche, mudflow, debris flow, etc)
- [ ] Hydro-Meteorological: Wave action
- [ ] Hydro-Meteorological: Storm
- [ ] Hydro-Meteorological: Extreme temperature
- [ ] Hydro-Meteorological: Fog
- [ ] Hydro-Meteorological: Drought
- [ ] Hydro-Meteorological: Wild fire
- [ ] Hydro-Meteorological: Glacial lake outburst (flood)
- [ ] Biological: Airborne diseases
- [ ] Biological: Waterborne diseases
- [ ] Biological: Vector-borne diseases
- [ ] Biological: Food-borne outbreaks
- [ ] Biological: Insect infestation
- [ ] Biological: Animal diseases
- [ ] Biological: Plant diseases
- [ ] Biological: Aeroallergens
- [ ] Biological: Antimicrobial resistant microorganisms
- [ ] Biological: Animal-human contact (snakes, spiders, etc)
- [ ] Extraterrestrial impact (airburst, meteorite, etc)
- [ ] Extraterrestrial: Space weather (energetic particles, geomagnetic storms, shockwave, etc)
- [ ] Human-induced: Industrial hazards
- [ ] Human-induced: Structural collapse (building collapse, dam/bridge failures. etc)
- [ ] Human-induced: Occupational hazards (mining, etc)
- [ ] Human-induced: Transportation (air, road, rail, water, space, etc)
- [ ] Human-induced: Explosions
- [ ] Human-induced: Fire
- [ ] Human-induced: Air pollution
- [ ] Human-induced: Infrastructure disruption (power outage, water supply, solid waste, waste water, telecommunication, etc)
- [ ] Human-induced: Cybersecurity
- [ ] Human-induced: Hazardous materials in air, soil, water
- [ ] Human-induced: Food contamination
- [ ] Human-induced: Acts of violence
- [ ] Human-induced: Armed conflicts
- [ ] Human-induced: Civil unrest
- [ ] Human-induced: Stampede
- [ ] Human-induced: Terrorism (Chemical, biological, radiological, nuclear, explosives, etc)
- [ ] Human-induced: Financial crises (hyperinflation, currency crisis)
- [ ] Environmental: Erosion
- [ ] Environmental: Deforestation
- [ ] Environmental: Salinization
- [ ] Environmental: Sea level rise
- [ ] Environmental: Desertification
- [ ] Environmental: Wetland loss/degradation
- [ ] Environmental: Glacier retreat/melting
- [ ] Environmental: Sand encroachment
- [ ] Other (please specify)

* A4.3 Please choose the **top 5** hazards that are most frequently dealt with by your center?

- [ ] Geophysical: Earthquake
- [ ] Geophysical: Tsunami
- [ ] Geophysical: Geophysical-trigger Mass movement (landslide, rock fall, subsidence, etc)
- [ ] Geophysical: Liquefaction
- [ ] Geophysical: Volcanic activity
- [ ] Hydro-Meteorological: Flood
- [ ] Hydro-Meteorological: Hydro-meteorological-trigger Mass movement (landslide, avalanche, mudflow, debris flow, etc)
- [ ] Hydro-Meteorological: Wave action
- [ ] Hydro-Meteorological: Storm
- [ ] Hydro-Meteorological: Extreme temperature
- [ ] Hydro-Meteorological: Fog
- [ ] Hydro-Meteorological: Drought
- [ ] Hydro-Meteorological: Wild fire
- [ ] Hydro-Meteorological: Glacial lake outburst (flood)
- [ ] Biological: Airborne diseases
- [ ] Biological: Waterborne diseases
- [ ] Biological: Vector-borne diseases
- [ ] Biological: Food-borne outbreaks
- [ ] Biological: Insect infestation
- [ ] Biological: Animal diseases
- [ ] Biological: Plant diseases
- [ ] Biological: Aeroallergens
- [ ] Biological: Antimicrobial resistant microorganisms
- [ ] Biological: Animal-human contact (snakes, spiders, etc)
- [ ] Extraterrestrial impact (airburst, meteorite, etc)
- [ ] Extraterrestrial: Space weather (energetic particles, geomagnetic storms, shockwave, etc)
- [ ] Human-induced: Industrial hazards
- [ ] Human-induced: Structural collapse (building collapse, dam/bridge failures. etc)
- [ ] Human-induced: Occupational hazards (mining, etc)
- [ ] Human-induced: Transportation (air, road, rail, water, space, etc)
- [ ] Human-induced: Explosions
- [ ] Human-induced: Fire
- [ ] Human-induced: Air pollution
- [ ] Human-induced: Infrastructure disruption (power outage, water supply, solid waste, waste water, telecommunication, etc)
- [ ] Human-induced: Cybersecurity
- [ ] Human-induced: Hazardous materials in air, soil, water
- [ ] Human-induced: Food contamination
- [ ] Human-induced: Acts of violence
- [ ] Human-induced: Armed conflicts
- [ ] Human-induced: Civil unrest
- [ ] Human-induced: Stampede
- [ ] Human-induced: Terrorism (Chemical, biological, radiological, nuclear, explosives, etc)
- [ ] Human-induced: Financial crises (hyperinflation, currency crisis)
- [ ] Environmental: Erosion
- [ ] Environmental: Deforestation
- [ ] Environmental: Salinization
- [ ] Environmental: Sea level rise
- [ ] Environmental: Desertification
- [ ] Environmental: Wetland loss/degradation
- [ ] Environmental: Glacier retreat/melting
- [ ] Environmental: Sand encroachment
- [ ] Other (please specify)

* A4.4 Which of the following activities and events have your center attended so far? Check all that apply.

- [ ] WHO EOC-NET Global
- [ ] WHO Regional PHEOC-NET (AFR)
- [ ] Regional PHEOC simulation exercise
- [ ] Global Pandemic Response Simulation Exercise
- [ ] Regional PHEOC training
- [ ] Staff trained as trainers and deployed to support other countries
- [ ] GOARN Training Workshop
- [ ] Rapid response team training
- [ ] Other (please specify)

* A4.5 Which of the above mentioned activities and events have your center benefited most from? Please select 1 or 2 activities.

- [ ] WHO EOC-NET Global
- [ ] WHO Regional PHEOC-NET (AFR)
- [ ] Regional PHEOC simulation exercise
- [ ] Global Pandemic Response Simulation Exercise
- [ ] Regional PHEOC training
- [ ] Staff trained as trainers and deployed to support other countries
- [ ] GOARN Training Workshop
- [ ] Rapid response team training
- [ ] Other (please specify)

* A4.6 Which types of exercises are most welcomed in your center?

- [ ] Orientation exercises (e.g. seminars, workshops)
- [ ] Table-top exercises
- [ ] Use of gaming (e.g. online video)
- [ ] Drills
- [ ] Functional exercises
- [ ] Full-scale exercises

* A4.7 Which training subjects are most needed in your center? (Select all that apply)

- [ ] Risk/hazard vulnerability analysis
- [ ] Multi-sectoral coordination
- [ ] PHEOC concept of operations
- [ ] IMS in action
- [ ] Planning
- [ ] Operations
- [ ] Communication
- [ ] Financial planning
- [ ] Information management
- [ ] Logistics
- [ ] Information and communication technology
- [ ] Other (please specify)

Do you have any other inputs or comments for the questions on this page?
(e.g if any answer exceeded the character limits, or if you have any other relevant comments, feel free to put them here.)

[Part A] Institutional Information

- A5. Individuals involved in your PHEOC

* A5.1a Please indicate the number of staff by employment type
(Enter 0 if you don't have any staff under any certain choice.)

Routine staff

Surge staff

Other staff

A5.1b Please specify the employment type of "Other staff" in Question A5.9a.
(Only applicable if you entered more than 0 in the "Other staff" choice.)

* A5.2 Please indicate all of the positions in the PHEOC and number of staff by each position.
(List in the format: Position [number], e.g. PHEOC Director [1].)

* A5.3 Are roles and responsibilities of each position clearly defined and understood in your center?

- Yes
- Somewhat
- No

* A5.4 Are there any other key positions that are not filled at your center?

- Yes
- Somewhat
- No

* A5.5 Do you have a job rotation for each positions?

- Yes
- No

* A5.6 Do you have a roster of trained staffs for each IMS function?

- Yes
- No

* A5.7 Do you have a clearly predefined roles and responsibilities for staff of the different IMS positions that can be adapted to context.

- Yes
- No

* A5.8 Please indicate the number of routine staff by age group
(Enter 0 if you don't have any staff under certain choice.)

< 20

20-24

25-34

35-44

45-54

55-64

> 65

* A5.10 Please indicate average working hours per week

- ◯ <20
- ◯ 21-30
- ◯ 31-40
- ◯ 41-50
- ◯ >50

Do you have any other inputs or comments for the questions on this page?
(e.g if any answer exceeded the character limits, or if you have any other relevant comments, feel free to put them here.)

This concludes Part A of the Institutional Survey.
Please proceed to next page to complete Part B and C.

[Part B] Legal framework, plans, procedures and infrastructures

In Part B, you will be asked to review the key items of legal authority, policy group, steering committee, planning, detailed implementation, physical infrastructure, information and communication technology infrastructure, information system and data standards, human resource, training, evaluation and funding issues involved in the PHEOC implementation and their impact on the performance of the PHEOC. Relevant personnel responsible for specific areas of work should be involved in completing the survey.

From scale 1-5, please check how you would rate the implementation of following items:
[0] - It has not been implemented / has no impact
[1] - To a very small extent
[2] - To a small extent
[3] - To a moderate extent
[4] - To a great extent
[5] - To a very great extent
[N/A] - Does not apply

* B1. Legal authority

	To what extent was the item implemented?	To what extent does the item have an impact on PHEOC's performance?
B1.1 Legal authority for PHEOC established		
B1.2 Government commitment has been secured		
B1.3 Public health emergencies are recognised as potential national disasters		

* B2. Policy group

	To what extent was the item implemented?	To what extent does the item have an impact on PHEOC's performance?
B2.1 A policy group to provide policy guidance to PHEOC has been established		

* B3. Steering committee

	To what extent was the item implemented?	To what extent does the item have an impact on PHEOC's performance?
B3.1 A steering committee of PHEOC stakeholders has been established for the planning and development of PHEOC		
B3.2 Hazards, vulnerabilities and resulting risks have been identified and prioritized		
B3.3 PHEOC objectives have been developed		

* B4. Planning

	To what extent was the item implemented?	To what extent does the item have an impact on PHEOC's performance?
B4.1<br>An all-hazards national public health emergency management plan, addressing priority risks, has been developed and approved		
B4.2<br>Emergency Response Plan for the health sector is in place		
B4.3<br>Response plans detail roles and responsibilities for MoH and other response agencies, sectors and jurisdiction at various levels in the response organization, including private sector and NGO organizations		
B4.4<br>Response plans describe scaled levels of response with resource requirements for each level and procedures for acquiring additional resources		
B4.5<br>Response plans detail the notification, reporting, engagement and coordination requirements		
B4.6<br>Response plans contain information about laboratories including: 1) Contact information; 2) Types, e.g. biosecurity level, locations, business hours and links to MoH surveillance systems; 3) Types of specimens or samples tested; 4) Types of testing provided; 5) Standard operating procedures for collection, packaging, shipping and maintaining chain of custody of specimens and samples		
B4.7<br>Response Plans contain verified location, contact and emergency response information for: 1) Hospitals, clinics and treatment centres; 2) Points of entry; 3) Pharmacies; 4) NGO's in-country; 5) Public health units; 6) Social services offices; 7) Shelters; 8) Partner government agencies; 9) IHR Focal Point and WHO IHR contact point; 10) Other subnational PHEOCs or National coordination centre;		
B4.8<br>Response plans provide SOPs for coordinating with law enforcement and national security agencies		
B4.9<br>The center has developed hazard specific plans based on risk assessment		

Do you have any other inputs or comments for the questions on this page?
(e.g if there's anything you would want to add for any item on this page, or if you have any relevant comments, feel free to put them here.)

[Part B] Legal framework, plans, procedures and infrastructures

* B5. Implementing

	To what extent was the item implemented?	To what extent does the item have an impact on PHEOC's performance?
B5.1<br>A clear operational structure based on the IMS and comprising (i) Management, (ii) Operations, (iii) Planning, (iv) Logistics and (v) Finance and Administration functions is in place		
B5.2<br>Staff, trained in emergency response management and mission-critical public health activities and objectives, are available to fill key PHEOC roles at all times		
B5.3<br>A roster of incident managers is maintained		
B5.4<br>The PHEOC manager has been appointed		
B5.5<br>The information manager has been appointed		
B5.6<br>The PHEOC has the capability to prepare public health alerts		
B5.7<br>The PHEOC has the capability to conduct web surveillance to detect and correct of rumours, public and interest group concerns and media misinformation		
B5.8<br>Infrastructure, personnel and procedures are in place sufficient to support IHR (2005) notification requirements (surveillance, detection, reporting, IHR focal point)		
B5.9<br>The PHEOC has the capability to produce and share a common operational picture		
B5.10<br>The PHEOC has the capability to direct and support logistical operations for the acquisition, storage, transportation and delivery of PPE, medical equipment, pharmaceuticals, laboratory supplies and medical countermeasure supplies for public health emergency responses in country		
B5.11<br>Administrative policy has been formulated to support emergency contracting, hiring, procurement and management of donor funds		

	To what extent was the item implemented?	To what extent does the item have an impact on PHEOC's performance?
B5.12<br>PHEOC plans (EOC plan and CONOPS) have been approved		
B5.13<br>The PHEOC plan/handbook for staff includes: 1) A concept of operations; 2) Map of the PHEOC workstations, rooms and inventories of equipment; 3) Routine staffing requirements; 4) Standard operating procedures; 5) Forms and templates for data collection, reporting, briefing, etc; 6) Documentation and records management processes; 7) Role descriptions and job aids for PHEOC functional positions; 8) Response levels and thresholds; 9) Activation, scaling, deactivation thresholds and procedures; 10) Contact information for key officials and PHEOC personnel; 11) Notification and communication protocols with host agency, response organizations and partner agencies		

* <u>B6. Plans and procedures</u>

	To what extent was the item implemented?	To what extent does the item have an impact on PHEOC's performance?
B6.1<br>Procedures and equipment are in place to establish and maintain communications between the IHR Focal Point and WHO Regional and Headquarters offices		
B6.2<br>Where relevant, hazard-specific response annexes have been developed to address chemical, infectious disease, radiological or food and water safety threats		
B6.3<br>Procedures in place for credentialing and permitting access for health professionals from other jurisdictions to operate in-country		
B6.4<br>The PHEOC has a Business Continuity Plan (Continuity of Operations Plan) which includes: 1) Priority functions that need to be maintained; 2) Key personnel that are needed to implement the plan; 3) Alternative/backup PHEOC site(s) and relocation plans; 4) Records and data management procedures; 5) Processes for maintaining critical external communications; 6) Activation, notification and deactivation procedures		
B6.5<br>The PHEOC has a communications plan for emergency public information and warning that outlines: 1) Triggers for issuing information to general public or specific audiences; 2) Message templates for priority threats; 3) Roles and responsibilities for communications staff; 4) Procedures for developing and approving new messages; 5) Procedures for issuing risk communications using traditional media, official social media accounts and agency website posting; 6) Process for developing linguistically and culturally appropriate messages; 7) Approval authorities for external messages; 8) Deactivation and demobilization plans describe procedures for notification, closing procedures, debriefings, records management, restoring and repatriating staff and supplies to pre-incident levels or to regular roles		

* B7. Physical infrastructure

	To what extent was the item implemented?	To what extent does the item have an impact on PHEOC's performance?
B7.1<br>A PHEOC suitable facility is available		
B7.2<br>A multi-use facility can be converted in one hour to an adequate operational PHEOC		
B7.3<br>A suitable facility has been acquired but not yet developed as an operational PHEOC		
B7.4<br>The PHEOC meets requirements for accessibility, security, structural integrity and resistance to natural and human generated hazards		
B7.5<br>The PHEOC has adequate space for the all expected PHEOC functions, private meetings, surge staff, secure communications, IT equipment and support personnel		
B7.6<br>The PHEOC has audiovisual functionality to project operational, contextual and event status information, tested and maintained		
B7.7<br>The PHEOC has sufficient potable water supply and adequate water to address sanitary requirements		
B7.8<br>The PHEOC has structural maintenance, janitorial and waste removal services		
B7.9<br>The PHEOC has toilet and sanitary facilities scaled for the expected occupancy		
B7.10<br>The PHEOC has approved quantity of first aid supplies an approved fire suppression system and/or equipment		
B7.11<br>The PHEOC has a staff evacuation plan		
B7.12<br>The PHEOC has security measures to control access		
B7.13<br>The PHEOC has a backup site that can be activated if the primary site becomes untenable		

* B8. Information and communication technology (ICT) infrastructure

	To what extent was the item implemented?	To what extent does the item have an impact on PHEOC's performance?
B8.1 The PHEOC has sufficient computer workstations with necessary application software loaded and tested.		
B8.2 PHEOC servers and backups, with needed applications are maintained and routinely tested		
B8.3 The PHEOC has sufficient tested telephonic and/ or interoperable radio communications for every workstation and meeting space, with spares.		
B8.4 There is tested web or video conferencing equipment in a private meeting space.		
B8.5 There are sufficient printers, copiers, fax machines and scanners are maintained and functional.		
B8.6 There is sufficient quantity of electricity including backup capacity (generator and fuel, UPS for critical data storage and processing)		
B8.7 The facility has an HVAC system sufficient to maintain comfort for occupants and keep IT equipment cool.		

Do you have any other inputs or comments for the questions on this page?
(e.g if there's anything you would want to add for any item on this page, or if you have any relevant comments, feel free to put them here.)

[Part B] Legal framework, plans, procedures and infrastructures

* B9. Information systems and data standards

	To what extent was the item implemented?	To what extent does the item have an impact on PHEOC's performance?
B9.1 The PHEOC has the capability to receive, analyze, display, report and share reports of reportable and unusual diseases and health conditions from: 1) public and private sector healthcare providers and facilities; 2) sub-national offices and units; 3) veterinary and animal health sources; 4) points of entry; 5) NGOs; 6) Other national governments and international agencies; 7) Other arms and branches of government and Community based sources		
B9.2 The PHEOC has the capability to collect, process and share field epidemiological and other investigational data (including (i) receiving, aggregating and analyzing field data, and (ii) visualization of epidemiological. information and timely preparation of reports in standardized format for decision making and sharing with partners)		
B9.3 The PHEOC is linked to a national surveillance information structure for monitoring and responding to priority risks.		
B9.4 The PHEOC has the capability to receive and share public health laboratory data related to outbreaks and events		
B9.5 The PHEOC has the capability to receive, produce and share integrated surveillance information containing epidemiological findings and laboratory results at individual and aggregated levels		
B9.6 The PHEOC has the capability to provide data analytic support for other events of public health interest (e.g: mass gatherings)		
B9.7 The PHEOC has the capability to produce geospatial information such as maps and other visualizations from common operational datasets		
B9.8 The PHEOC has the capability to ascertain the status and report key external partner/resource information such as hospital bed availability, treatment centres, laboratories etc.		

	To what extent was the item implemented?	To what extent does the item have an impact on PHEOC's performance?
B9.9<br>The PHEOC has the capability to monitor the status and needs of deployed field teams and other responder personnel including assisting international support and NGOs		
B9.10<br>The PHEOC has the capability to display contextual operational information such as population distribution, administrative and political boundaries, transportation infrastructure, hydrology and elevations		
B9.11<br>The PHEOC datasets include identification of information necessary to develop a common operational picture for priority risks		
B9.12<br>The PHEOC can access, display and track status information regarding affected persons and vulnerable populations		
B9.13<br>The PHEOC has the capability to monitor and account for all resources utilized in a response		
B9.14<br>The PHEOC has the capability to track and display the status of tasks and objectives		
B9.15<br>Staff filling IMS functional roles are trained to use relevant software		

* B10. Human resources

	To what extent was the item implemented?	To what extent does the item have an impact on PHEOC's performance?
B10.1<br>The PHEOC has designated staffing to manage the facility		
B10.2<br>The PHEOC has designated routine staff (eg. PHEOC manager, operations lead, planning officer, communication officer, logistics officer etc)		
B10.3<br>Technical personnel are available for the operation, maintenance and repair of audio-visual, telecommunications and computer equipment		
B10.4<br>There are personnel trained in emergency management and PHEOC standard operating procedures		
B10.5<br>There is sufficient trained staff to activate the PHEOC on short notice		
B10.6<br>The PHEOC has the capability to identify and contact a roster of trained personnel, equipment and supplies for deployment to field sites responders (e.g. rapid response teams)		

* B11. Training and exercising

	To what extent was the item implemented?	To what extent does the item have an impact on PHEOC's performance?
B11.1<br>The PHEOC has a dedicated training program based on a training needs assessment for incident management personnel that addresses staff roles during response operations; utilization of communications and data processing equipment and software; and hazard-specific response knowledge		
B11.2<br>The PHEOC has a comprehensive, progressive exercise program for all staff and partners, national and NGO agencies and produces evaluation reports that identify corrective actions required		

* <u>B12. Monitoring and evaluating</u>

	To what extent was the item implemented?	To what extent does the item have an impact on PHEOC's performance?
B12.1 The PHEOC training and exercise programs are primary components of a performance monitoring and evaluation system focused on continuous improvement of public health emergency management capability and effectiveness		

* <u>B13. Costing, funding and sustaining</u>

	To what extent was the item implemented?	To what extent does the item have an impact on PHEOC's performance?
B13.1 The PHEOC plan includes an itemized schedule of costs		
B13.2 There is funding plan and funding mechanism to support the PHEOC		
B13.3 Funds are available to develop and sustain the PHEOC		

Do you have any other inputs or comments for the questions on this page?
(e.g if there's anything you would want to add for any item on this page, or if you have any relevant comments, feel free to put them here.)

This concludes Part B of the Institutional Survey.
Please proceed to next page and complete the last part.

[Part C] Case study of COVID-19

- C1. Documenting the timeliness of COVID-19 response

This part asks for the timeline of PHEOC's activities during the COVID-19 epidemic. Personnel involved in the COVID-19 response should be consulted in providing information to complete this part.
Please include time [HH:MM] and dates [DD-MM-YYYY] in your answers.

* C1.1 What is the timeline for each of the operations mode (from watch to alert, to response)?
Please include time [HH:MM, DD-MM-YYYY] in your answers.

* C1.2 When was the PHEOC activated for COVID-19 management (preparedness), at what criteria?
Please include time [HH:MM, DD-MM-YYYY] in your answers.

* C1.3 To which level was the PHEOC activated?

* C1.4 Have you conducted rapid risk assessment in order to determine the activation level?

Yes No

C1.5 (If answered yes in C1.4) How long did it take to conduct the rapid risk assessment?

* C1.6 How long did it take for the initial activation of the PHEOC?

* C1.7 When did you establish the incident management system (IMS) structure?

Please select the date below

Date

MM/DD/YYYY

* C1.8 Have you increased or decreased the activation level of PHEOC since the start of COVID-19?

Increased Decreased Neither

* C1.9 When did you update the critical information requirements to monitor the situation?

Please select the date below

Date

MM/DD/YYYY

* C1.10 When did you develop and share with senior management the Spotrep for COVID?

Please select the date below

Date

MM/DD/YYYY

Do you have any other inputs or comments for the questions on this page?
(e.g if any answer exceeded the character limits, or if you have any other relevant comments, feel free to put them here.)

[Part C] Case study of COVID-19

- C1. Documenting the timeliness of COVID-19 response (continued)

* C1.11 How long did it take to identify the first case of COVID-19 in the country?

* C1.12 When did you identify the first case of COVID-19 in the country?

Please select the date below

Date

MM/DD/YYYY

* C1.13 When did the IHR NFP notify WHO of the cases?

Please select the date below

Date

MM/DD/YYYY

* C1.14 When did you issue the first SitRep?

Please select the date below

Date

MM/DD/YYYY

* C1.15 When did you activate hotline to receive alerts?

Please select the date below

Date

MM/DD/YYYY

* C1.16 When was the preparedness plan developed for COVID-19?

Please select the date below

Date

MM/DD/YYYY

* C1.17 When was the first incident action plan developed?

Please select the date below

Date

MM/DD/YYYY

* C1.18 How long did it take to develop the first incident action plan?

* C1.19 Do the watch staff/team continue to monitor other events apart from COVID-19?

- ◯ Yes
- ◯ No

* C1.20 Were there other events required PHEOC activation during this period of time? Please specify.

* C1.21 Please describe how the PHEOC manage multiple events.

Do you have any other inputs or comments for the questions on this page?
(e.g if any answer exceeded the character limits, or if you have any other relevant comments, feel free to put them here.)

[Part C] Case study of COVID-19

- C2. Intervention and response

In this section, you will be asked to indicate the type and timeline of COVID-19 transmission scenarios observed in your responsible country.

Four transmission scenarios will be reviewed:

1) No cases

2) One or more cases, imported or locally acquired (Sporadic cases)

3) Experiencing cases clusters in time, geographic location, or common exposure (Clusters of cases)

4) Experiencing larger outbreaks of local transmission (Community transmission)

2.1 No cases scenario: Your country has no COVID-19 cases observed during a time range.

* C2.1a Did your country observe a No cases scenario?

- ◯ Yes, there are no cases observed in the early stage of the epidemic ⟶ Jump to C2.1b
- ◯ Yes, there are no cases observed up to the date of this survey ⟶ Skip to C2.1c

[Part C] Case study of COVID-19

- C2. Intervention and response (continued)

* C2.1b When did the No cases scenario end in your country:

Please select the date below

Date

MM/DD/YYYY

[Part C] Case study of COVID-19

- C2. Intervention and response (continued)

* C2.1c With <u>No cases</u> scenario, have you taken the following interventions? (Select all that apply)

- [ ] Set up screening and triage protocols at all points of access to the health system, including primary health centres, clinics, hospital emergency units, and ad hoc community settings.
- [ ] Set up COVID-19 telephone hotline and referral system to refer patients to the appropriate destination for clinical assessment and/or testing as per local protocol.
- [ ] Set up COVID-19 designated wards in health facilities.
- [ ] Conduct active case finding, contact tracing and monitoring, quarantine of contacts, and isolation of suspected cases.
- [ ] None of the above mentioned interventions were taken.
- [ ] Other (please specify all the other interventions you have taken)

Do you have any other inputs or comments for the questions on this page?
(e.g if any answer exceeded the character limits, or if you have any other relevant comments, feel free to put them here.)

[Part C] Case study of COVID-19
- C2. Intervention and response (continued)

2.2 Sporadic cases scenario: Your country observed one or more cases, imported or locally acquired.

* C2.2a Did your country observe a Sporadic cases scenario?

- ◯ Yes ⟶ Jump to C2.2b
- ◯ No ⟶ Skip to C2.3a

[Part C] Case study of COVID-19

- C2. Intervention and response (continued)

* C2.2b Please indicate the starting and ending date of Sporadic cases scenario observed in your country.

Starting date

Date

MM/DD/YYYY

Ending date

Date

MM/DD/YYYY

* C2.2c With Sporadic cases scenario, have you taken the following interventions? (Select all that apply)

- [ ] Screen and triage at all points of access to the health system, including primary health centres, clinics, hospital emergency units, and ad hoc community settings.
- [ ] Care for all suspected and confirmed COVID-19 patients in isolation (or cohorting) according to disease severity and acute care needs for treatment at the COVID-19 designated treatment area
- [ ] Continue rapid and thorough contact tracing and quarantine of contacts.
- [ ] None of the above mentioned interventions were taken.
- [ ] Other (please specify all the other interventions you have taken)

Do you have any other inputs or comments for the questions on this page?
(e.g if any answer exceeded the character limits, or if you have any other relevant comments, feel free to put them here.)

[Part C] Case study of COVID-19

- C2. Intervention and response (continued)

2.3 <u>Clusters of cases</u> scenario: Your country observed cases clusters in time, geographic location, or common exposure.

* C2.3a Did your country observe a Clusters of cases scenario?

- ◯ Yes ⟶ Jump to C2.3b
- ◯ No ⟶ Skip to C2.4a

[Part C] Case study of COVID-19
- C2. Intervention and response (continued)

* C2.3b Please indicate the starting and ending date of Clusters of cases scenario observed in your country.

Starting date

Date

MM/DD/YYYY

Ending date

Date

MM/DD/YYYY

* C2.3c With Clusters of cases scenario, have you taken the following interventions? Select all that apply.

- [ ] Screen and triage at all points of access to the health system, including primary health centres, clinics, hospital emergency units, and ad hoc community settings.
- [ ] Care for all COVID-19 patients in the designated treatment area, according to disease severity and acute care needs according to the WHO recommendations.
- [ ] Surge by repurposing wards or ICUs into COVID-19 wards and hospitals.
- [ ] Where health facilities can no longer manage patients with mild or moderate disease, isolate patients who are not at high risk for severe disease (< 60 years of age, no co-morbid diseases) either in community facilities (e.g. stadium, gymnasium, hotel, or tent) with access to rapid health advice (i.e. via adjacent dedicated COVID-19 health post, telemedicine) or at home according to WHO guidance. If patient develops symptoms that may correspond to complications, ensure rapid referral to hospital.
- [ ] Plan for new structures to augment the health system based on the assumption that the number of cases will double every 3 to 7 days subject to the effectiveness of public health interventions.
- [ ] None of the above mentioned interventions were taken.
- [ ] Other (please specify all the other interventions you have taken)

Do you have any other inputs or comments for the questions on this page?
(e.g if any answer exceeded the character limits, or if you have any other relevant comments, feel free to put them here.)

[Part C] Case study of COVID-19

- C2. Intervention and response (continued)

2.4 Community transmission scenario: Your country observed larger outbreaks of local transmission.

* C2.4a Did your country observe a Community transmission scenario?

- ◯ Yes ⟶ Jump to C2.4b
- ◯ No ⟶ Skip to C3.1

[Part C] Case study of COVID-19
- C2. Intervention and response (continued)

* C2.4b Please indicate the starting and ending date of Clusters of cases scenario observed in your country.

Starting date

Date

MM/DD/YYYY

Ending date

Date

MM/DD/YYYY

* C2.4c With community transmission scenario, have you taken the following interventions? (Select all that apply)

- [ ] Screen and triage at all points of access to the health system, including primary health centres, clinics, hospital emergency units, and ad hoc community settings.
- [ ] Care for all suspected and confirmed COVID-19 patients in the designated treatment area, according to disease severity and acute care needs according to the WHO recommendations.
- [ ] Surge the health system with new structures established for care delivery, including rapid extension of designated hospitals to care for COVID-19 patients.
- [ ] New hospitals or temporary structures can serve to augment COVID-19 patient care or essential health services, depending on national strategy.
- [ ] Referrals adopt a “hub and spoke” model, with a central COVID-19 referral facility and all other health facilities in each geographical area referring patients to the nearest centre.
- [ ] Manage all mild and low- to moderate risk patients with confirmed disease in designated community facilities (e.g. stadium, gymnasium, hotel or tent) with access to rapid health advice (i.e. via adjacent dedicated COVID-19 health post, telemedicine) or at home according to WHO guidance and national or subnational capacity. If patient develops symptoms that may correspond to severe disease or complications, ensure rapid referral to hospital.
- [ ] Depending on testing strategy and capacity, mild and moderate patients may not be tested, and advised to self-isolate either in cohorted community facilities or at home.
- [ ] None of the above mentioned interventions were taken.
- [ ] Other (please specify all the other interventions you have taken)

Do you have any other inputs or comments for the questions on this page?
(e.g if any answer exceeded the character limits, or if you have any other relevant comments, feel free to put them here.)

[Part C] Case study of COVID-19

- C3. Supporting the COVID-19 Response

* C3.1 Dose the PHEOC have enough space to accommodate surge staff in COVID-19 preparedness and response?

- ◯ Yes
- ◯ No

* C3.2 How well would you rate the information system in supporting the COVID-19 preparedness and response?

- ◯ Not helpful at all
- ◯ Not supportive enough
- ◯ Works fine, better than nothing
- ◯ Quite helpful but can be improved
- ◯ Very satisfactory

Congratulations!

You have completed all questions for the Institutional Survey.
Please click "Submit" to save your answers.

Thank you very much for your participation.
Have a nice day.

If you have any other comments about the questions in this survey, please leave below:
