# Supplementary material for: Implementing public health emergency operations centres according to an international framework in Ethiopia, Nigeria, and Senegal: Best practices and achievements, 2021
Source: PLOS Glob Public Health. 2026 Apr 27;6(4):e0006211. doi: 10.1371/journal.pgph.0006211 (PMC13119900; doi:10.1371/journal.pgph.0006211)
Supplement: S2 Text — (PDF) [file pgph.0006211.s002.pdf]

Welcome to the Individual Capacity Survey.

We are conducting this survey to document the development and implementation of your PHEOC, its role in responding to the COVID-19 pandemic, as well as to address the gaps and needs in PHEOCs best practices guidance, and to provide policy recommendations for implementing and strengthening PHEOC to promote public health emergency preparedness and response in Africa region.

This study is being conducted by the World Health Organization.

There are two sets of questionnaires for this study, the institutional survey and the individual capacity survey.

This is the Individual Capacity Survey. This survey consists of three parts: Part A (Knowledge and concepts for PHEOC Operation), Part B (Knowledge, Skills and Abilities required for specific PHEOC functions) and Part C (Core competencies for public health emergency). You will need to complete all three parts of the survey.

If you are eligible and decide to participate, you shall proceed to complete the questionnaire below. You will be asked about questions related to knowledge, skills and abilities required for the PHEOC's daily operation and management. Please note that these questions are not intended to rate your performance and provide feedback to your supervisor. We collect these information solely to analyze the implementation, strength and gaps of your PHEOC, so as to document the best practices and reflect future improvement to strengthen the effectiveness of the PHEOC for public health emergency response. The result of this survey will not be shared with your supervisor and will remain confidential. Some basic information about your personal background might be collected, including your name, position, etc. Some of the participants will be further interviewed or invited to group discussions after the questionnaire survey regarding the same topics.

In this regard, please answer the questions based on your honest situation. Please do not select randomly or select 5 for all the self-rated questions.

There is a minimal risk of breach of confidentiality, which we have plans to avoid with full effort.

The following provides more information about the study and the survey.

1. What is the purpose of this study?

This study aims to document the development and implementation of PHEOCs in Ethiopia, Nigeria and Senegal, and to provide best practices and policy recommendations for implementing and strengthening PHEOC to promote public health emergency preparedness and response in Africa region.

The study has three main objectives:

a) To document the implementation of PHEOCs based on best practice guidance;

b) To document the added value of PHEOC to COVID-19 preparedness and response;

c) To discuss the barriers and facilitators in PHEOCs development and implementation

2. Who are eligible for this study?

PHEOC staffs (routine and surge) and emergency responders are eligible to participate in this study.

3. What will be surveyed in this study?

Through questionnaire survey, interviews and group discussions, the implementation of your PHEOC will be documented, including policies, plans and procedures; organizational structure; human resources; information system and standards, communication technology and infrastructure, etc and its role in public health emergency response including COVID-19 pandemic.

There are two parts of surveys, an institutional survey and an individual capacity survey. The one you are taking now is the individual capacity survey, which mainly focus on human resource capacity and trainings.

Anything we collect in this study serves to better understand the overall implementation of the PHEOC, instead of anything personal to you.

4. How long will this study last?

Most of you will only be asked to complete this series of questionnaires. Some of you might be invited to participate in an interview or group discussion.

5. What are the risks of participating in this study?

There is a minimal risk of breach of confidentiality. The questions involve your basic information like name and position (to track the progress of research), and self-rated knowledge and capacity level in your work. The study team has procedures in place to help protect your privacy.

6. How will information about you be protected?

WHO policy on the use and sharing of data collected by WHO in Member States outside the context of public health emergencies will be followed (The WHO policy can be accessed from https://www.who.int/about/who-we-are/publishing-policies/data-policy).

Specific measures will be in place to ensure the highest precautions to protect your privacy and confidentiality. This includes:

- You will be assigned a study ID number which we will use to keep track of your information.
- The information we collect will be stored in a locked drive within the study investigator’s locked office.
- All information is entered into an encrypted, password protected computer.
- Only the study team members will have access to the data collected.
- You will not be identified in any report or publication about this study. Every effort will be made to keep research records private.

If you also participate in the in-depth interview afterwards, we will digitally audio record the interviews.

- The recorded files and transcripts will be stored on an encrypted, password protected computer and will be deleted at the end of the study.
- During the interview, you may ask to have the audio recorder turned off at

7. What are the benefits of participating in the study?
This study aims to address the gaps between the current implementation and the best practice of PHEOC. The result of this study could potentially improve the the effectiveness of your PHEOC for public health emergency response.

8. What if you want to stop before your part in the study is complete?
You can withdraw from this study at any time. The investigators also have the right to stop your participation at any time. This could be because you have failed to follow instructions, or because the entire study has been stopped.
If you withdraw or are withdrawn from this study all data collected up until the point of withdrawal will be retained, however no additional information will be collected unless you provide additional written permission for further data collection at the time of your withdrawal.

9. Will it cost you anything to be in this study?
It will not cost you anything to be in this study.

10. Will you be paid if you participate in this study?
No, you will not receive and financial support in participating the study.

11. What if you have questions about this study?
If you have questions about the study, complaints, concerns, or if a research-related injury occurs, you should contact the research team of this study.

Participant's Electronic Agreement

I have read the information provided above. I fully understand this study and its risks. I have asked all the questions I have at this time. I understand who to contact if I have further questions or complaints. I understand that I can withdraw from the study any time I want. I voluntarily agree to participate in this study.

PLEASE NOTE:
Since this is a web-based survey, we will not require your signature in this consent form.
Proceeding to fill out the questionnaires indicates that you CONSENT to voluntarily participate in this study and ACCEPT the possible risks.

Thank you for participating in this survey, your feedback is very important.

General information of survey participant

We collect these information to ease the tracking and analysis of the research.
Only the research team will have access to the information you provide below.
We are trying our best to protect your privacy.

* In which country do you work?

- ◯ Ethiopia
- ◯ Nigeria
- ◯ Senegal

* In what province do you work?

[ ]

* What is the name of your institution?

[ ]

* Your contact name:

[ ]

* Your position in the institution:

[ ]

* What is your highest education level?

- ◯ Less than high-school diploma
- ◯ High-school diploma
- ◯ Bachelor's degree
- ◯ Master's degree
- ◯ Doctoral degree

What is your gender?

- ◯ Female
- ◯ Male
- ◯ Other
- ◯ Prefer not to say

* What is your age group?

- Under 18
- 18-24
- 25-34
- 35-44
- 45-54
- 55-64
- 65+
- Prefer not to say

* Where did you work before working at the PHEOC? What was your position?

Organization

Position

* Date of filling this questionnaire

Please select the date below

Date

DD/MM/YYYY

[Part A] Knowledge and concepts for PHEOC Operation

* A1. Have you ever received formal trainings on understanding and operating PHEOCs?

- ◯ Yes, I have received trainings that provides me with sufficient knowledge and skills;
- ◯ Yes, I have attended relevant trainings but I don't consider them enough for my work;
- ◯ No, I have not received any kind of formal trainings related to PHEOC;

* A2. Please list the formal trainings related to emergency management and EOC operation you received?

* A3. What organizations are these trainings delivered by? Select all that was involved in designing and delivering the trainings.

- [ ] Ministry of Health
- [ ] World Health Organization
- [ ] African CDC
- [ ] National CDC
- [ ] US CDC
- [ ] Public Health England
- [ ] WAHO (West African Health Organization)
- [ ] Other (please specify)

* A4.1 What are the main sources of your knowledge about emergency management and EOC operation?

- [ ] Classroom-based courses
- [ ] Online e-learning courses
- [ ] Workshops
- [ ] EOC-NET meetings, publications, exercises
- [ ] PHEOC Train the trainers course
- [ ] PHEOC Train the trainers cascade courses
- [ ] Referred reading materials
- [ ] Participation in PHEOC planning and development of operating procedures
- [ ] Site and field assignments to learn from experience
- [ ] Participation in peer-to-peer learning, coaching, mentoring, and team-building
- [ ] Participation in exercises

* A4.2 Please rank these sources in the order you consider most helpful. (Put the sources you didn't receive knowledge from at the bottom.)

Classroom-based courses

Online e-learning courses

Workshops

EOC-NET meetings, publications, exercises

PHEOC Train the trainers course

PHEOC Train the trainers cascade courses

Referred reading materials

Participation in PHEOC planning and development of operating procedures

Site and field assignments to learn from experience

Participation in peer-to-peer learning, coaching, mentoring, and team-building

Participation in exercises

Do you have any other inputs or comments for the questions on this page?
(e.g if any answer exceeded the character limits, or if you have any other relevant comments, feel free to put them here.)

[Part A] Knowledge and concepts for PHEOC Operation

* **A5. Please select whether you agree or disagree with the following statements.**
Note that some statements might be false.

	Agree	Disagree
A5.1 A PHEOC must be part of a comprehensive programme of public health emergency preparedness, planning and capacity building.	○	○
A5.2 An emergency response plan (ERP) covers all the time period from the start of a public health emergency to after the emergency.	○	○
A5.3 The Incident Management System (IMS) generally has five functions: Management, Operation, Planing and Logistics, Finance and administration.	○	○
A5.4 In any PHEOCs, a policy group should be established to provide policy guidance and a steering committee should be formed for the planning and development of the PHEOC.	○	○
A5.5 The purpose of a PHEOC is coordination of information and resources.	○	○
A5.6 The PHEOC information system must be seamlessly integrated with other relevant national information systems.	○	○
A5.7 The information system of a PHEOC must be built upon existing initiatives and systems.	○	○

[Part A] Knowledge and concepts for PHEOC Operation

A6. Please select the answer to the following questions:

* A6.1 The size, type and scope of a PHEOC should be determined by its:

- [ ] Directors
- [ ] Policy Group
- [ ] Steering Committee
- [ ] Supervising Government Department
- [ ] Funding agency

* A6.2 Which of the following is the key to how multi-sectoral and multi-layer coordination will work at the strategic, operational and tactical levels?

- [ ] The legal mandate of the PHEOC
- [ ] The Emergency Response Plan of the PHEOC
- [ ] The Standard Operating Procedures of the PHEOC
- [ ] The Concept of operations of the PHEOC

* A6.3 Which of the following is a commonly recognized element of a comprehensive emergency management programme? (Select all that apply)

- [ ] Risk assessment
- [ ] Prevention and mitigation
- [ ] Preparedness
- [ ] Response
- [ ] Recovery

* A6.4 Which type of data are required in PHEOC?

- [ ] Event specific data
- [ ] Event management information
- [ ] Context data

[Part A] Knowledge and concepts for PHEOC Operation

* **A7. At the present time, how would you rate your ability in the following tasks?**
(1 = very limited, 5 = very good)

	1	2	3	4	5
A7.1 Determine the level of activation required for the emergency	◯	◯	◯	◯	◯
A7.2 Identify the best functional layout for a PHEOC based on operational requirements	◯	◯	◯	◯	◯
A7.3 Develop a staffing plan for the functions of the IMS	◯	◯	◯	◯	◯
A7.4 Identify ICT and data management needs	◯	◯	◯	◯	◯
A7.5 Perform assigned function in PHEOC with necessary ICT skills	◯	◯	◯	◯	◯
A7.6 Identify, key partners in inter-agency coordination	◯	◯	◯	◯	◯
A7.7 Access the PHEOC and its resources	◯	◯	◯	◯	◯
A7.8 Complete a template for action planning	◯	◯	◯	◯	◯
A7.9 Develop job action sheets for the main functions of the IMS	◯	◯	◯	◯	◯

This concludes Part A of the Individual Capacity Survey.
Please proceed to next page to complete Part B and C.

[Part B] Knowledge, Skills and Abilities required for specific PHEOC roles and functions

Thank you for completing Part A of the survey.

In Part B, you will be asked about the knowledge, skills and abilities that might be used to support specific PHEOC roles and functions, including: Policy, Management/Leadership, Operation, Planning, Communication, Logistic, Finance and administration.

You will only need to answer the questions relevant to your job mandate.

B1. Policy role

B1.0 Is your job mandate partly involved with PHEOC's *Policy* role?

◯ Yes ◯ No ◯ Not sure

Yes / Not sure ⟶ Jump to B1.1

No ⟶ Skip to B2.0

[Part B] Knowledge, Skills and Abilities required for specific PHEOC roles and functions

Please note that we are collecting these information solely to analyze the strength and gaps in your PHEOC's implementation, so as to document the best practices and reflect future improvement for your capacity building programs. The result of this survey will NOT be shared with your PHEOC supervisors.

In this regard, please answer the questions based on your honest situation. Please do NOT select randomly or select 5 for all the self-rated questions.

B1. Policy role

* **In general, how well do you think you can fulfill the following subjects?**
(1 = very poorly, 5 = very well)

	1	2	3	4	5
B1.1 Identify current health trends and gather information that can inform options for policies, programmes and services	○	○	○	○	○
B1.2 Recognize the value in having an incident management structure during an emergency situation	○	○	○	○	○
B1.3 Identify limits to legal knowledge, skill, and authority and identify key system resources, including legal advisors, for referring matters that exceed those limits	○	○	○	○	○
B1.4 Describe the legal authorities related to the distribution and dispensation of medical supplies	○	○	○	○	○
B1.5 Understand the effect of a state/federal public health emergency declaration on the authorities mentioned in B1.4	○	○	○	○	○

[Part B] Knowledge, Skills and Abilities required for specific PHEOC roles and functions

B2. Management/Leadership role

B2.0 Is your job mandate partly involved with PHEOC's *Management/Leadership* role?

◯ Yes ◯ No ◯ Not sure

Yes / Not sure ⟶ Jump to B2.1

No ⟶ Skip to B3.0

[Part B] Knowledge, Skills and Abilities required for specific PHEOC roles and functions

Please note that we are collecting these information solely to analyze the strength and gaps in your PHEOC's implementation, so as to document the best practices and reflect future improvement for your capacity building programs. The result of this survey will NOT be shared with your PHEOC supervisors.

In this regard, please answer the questions based on your honest situation. Please do NOT select randomly or select 5 for all the self-rated questions.

B2. Management/Leadership role

* **In general, how well do you think you can fulfill the following subjects?**
(1 = very poorly, 5 = very well)

	1	2	3	4	5
B2.1 Set and follow priorities, and maximize outcomes based on available resources	○	○	○	○	○
B2.2 Fulfill functional roles in response to a public health emergency	○	○	○	○	○
B2.3 Develop staff by providing opportunities for professional development like training, mentoring, peer advising, coaching, etc.	○	○	○	○	○
B2.4 Facilitate collaboration with internal and external emergency response partners	○	○	○	○	○
B2.5 Demonstrate advanced problem-solving skills under emergency conditions and adapt in response to changes (social, political, economic, and/or scientific change)	○	○	○	○	○
B2.6 Utilize staff and technology to maintain situational awareness and common operational picture	○	○	○	○	○
B2.7 Distinguish routine from urgent management information	○	○	○	○	○
B2.8 Classify information for internal and external audiences and distinguish the roles of staff involved in collecting and disseminating information for audiences (e.g. coordinator, public information officer, technology/IT departments, etc.)	○	○	○	○	○

	1	2	3	4	5
B2.9<br>Define the roles and responsibilities of public health personnel in a variety of public health emergencies and in the incident management system	○	○	○	○	○
B2.10<br>Categorize and evaluate potential threats and emergencies	○	○	○	○	○
B2.11<br>Demonstrate commitment to the safety of personnel by employing protective behaviours according to changing conditions, personal limitations and threats	○	○	○	○	○
B2.12<br>Describe the relationship between protective measures and behaviours and reduction of risk of injury or illness for personnel	○	○	○	○	○
B2.13<br>Employ practices to minimize exposure to agents and hazards during an emergency	○	○	○	○	○
B2.14<br>Know and act within the scope of national, state, tribal, and/or local statutory and regulatory authority during public health emergencies and through state and/or national declarations of emergency.	○	○	○	○	○
B2.15<br>Directs activities based on explicit authority, such as establishing incident objectives, strategies, and priorities.	○	○	○	○	○
B2.16<br>Involve decision-making and coordinating over all response	○	○	○	○	○

[Part B] Knowledge, Skills and Abilities required for specific PHEOC roles and functions

B3. Operations function

B3.0 Is your job mandate partly involved with PHEOC's *Operations* function?

- ◯ Yes
- ◯ No
- ◯ Not sure

Yes / Not sure ⟶ Jump to B3.1

No ⟶ Skip to B4.0

[Part B] Knowledge, Skills and Abilities required for specific PHEOC roles and functions

Please note that we are collecting these information solely to analyze the strength and gaps in your PHEOC's implementation, so as to document the best practices and reflect future improvement for your capacity building programs. The result of this survey will NOT be shared with your PHEOC supervisors.

In this regard, please answer the questions based on your honest situation. Please do NOT select randomly or select 5 for all the self-rated questions.

B3. Operations function

* **In general, how well do you think you can fulfill the following subjects?**
(1 = very poorly, 5 = very well)

	1	2	3	4	5
B3.1 Interpret and communicate procedures in emergency operations plans and other procedures for operations of technical health expertise (eg. surveillance, case management, infection prevention and control, risk communication etc.)	○	○	○	○	○
B3.2 Recognize information potentially relevant to the identification and control of an emergency and report it through the chain of command	○	○	○	○	○
B3.3 Know and manage or apply decontamination or disinfection procedures as necessary	○	○	○	○	○
B3.4 Use information technology in accessing, collecting, analyzing, using, maintaining, and disseminating data and information, and use informatics standards	○	○	○	○	○
B3.5 Apply ethical principles in accessing, collecting, analyzing, using, maintaining, and disseminating data and information	○	○	○	○	○
B3.6 Determine quantitative and qualitative data and information	○	○	○	○	○
B3.7 Collect, analyze and interpret data to determine validity and reliability	○	○	○	○	○
B3.8 Practice process improvement	○	○	○	○	○
B3.9 Establish, train and prepare RRTs at national and sub national levels	○	○	○	○	○
B3.10 Alert the PHEOC manager any potential treat and prepare SPOTREP to inform the leadership	○	○	○	○	○
B3.11 Lead the watch staff to constantly monitors and triage information on public health events by facilitating the collection, organization, analysis, distribution, and archiving of information	○	○	○	○	○

[Part B] Knowledge, Skills and Abilities required for specific PHEOC roles and functions

B4. Planning function

B4.0 Is your job mandate partly involved with PHEOC's *Planning* function?

- ◯ Yes
- ◯ No
- ◯ Not sure

Yes / Not sure ⟶ Jump to B4.1

No ⟶ Skip to B5.0

[Part B] Knowledge, Skills and Abilities required for specific PHEOC roles and functions

Please note that we are collecting these information solely to analyze the strength and gaps in your PHEOC's implementation, so as to document the best practices and reflect future improvement for your capacity building programs. The result of this survey will NOT be shared with your PHEOC supervisors.

In this regard, please answer the questions based on your honest situation. Please do NOT select randomly or select 5 for all the self-rated questions.

B4. Planning function

* **In general, how well do you think you can fulfill the following subjects?**
(1 = very poorly, 5 = very well)

	1	2	3	4	5
B4.1 Coordinate to the development and implementation of the organizational strategic plan, emergency operations plans including hazard specific plan and incident action plan	○	○	○	○	○
B4.2 Gather appropriate information for evaluating policies, programmes and services	○	○	○	○	○
B4.3 Apply strategies for continuous quality improvement	○	○	○	○	○
B4.4 Verify the credibility of information sources	○	○	○	○	○
B4.5 Use analytical tools to analyze information and recommend specific actions	○	○	○	○	○
B4.6 Conduct exercises to test plans and procedures and systems	○	○	○	○	○
B4.7 Develops corrective action plans following after action review and post exercise and monitor implementation	○	○	○	○	○
B4.8 Prepares the demobilization plan and monitors implementation	○	○	○	○	○
B4.9 Maintains records of response activities to support accountability	○	○	○	○	○
B4.10 Maintain situational awareness and common operational picture	○	○	○	○	○

	1	2	3	4	5
B4.11<br>Maintains records of response activities and support accountability through tracking of implementation of incident action plan and conduct of monitoring and evaluation	○	○	○	○	○
B4.12<br>Use information technology in accessing, collecting, analyzing, using, maintaining, and disseminating data and information, and use informatics standards	○	○	○	○	○
B4.13<br>Apply ethical principles in accessing, collecting, analyzing, using, maintaining, and disseminating data and information	○	○	○	○	○
B4.14<br>Determine quantitative and qualitative data and information	○	○	○	○	○

[Part B] Knowledge, Skills and Abilities required for specific PHEOC roles and functions

B5. Communication role

B5.0 Is your job mandate partly involved with PHEOC's *Communication* role?

- ◯ Yes
- ◯ No
- ◯ Not sure

Yes / Not sure ⟶ Jump to B5.1

No ⟶ Skip to B6.0

[Part B] Knowledge, Skills and Abilities required for specific PHEOC roles and functions

Please note that we are collecting these information solely to analyze the strength and gaps in your PHEOC's implementation, so as to document the best practices and reflect future improvement for your capacity building programs. The result of this survey will NOT be shared with your PHEOC supervisors.

In this regard, please answer the questions based on your honest situation. Please do NOT select randomly or select 5 for all the self-rated questions.

B5. Communication role

* **In general, how well do you think you can fulfill the following subjects?**
(1 = very poorly, 5 = very well)

	1	2	3	4	5
B5.1 Differentiate between risk communication and emergency crisis communication	○	○	○	○	○
B5.2 Prepare and deliver messages using the principles and guidelines of crisis and risk communication	○	○	○	○	○
B5.3 Demonstrate cultural sensitivity as essential in communicating with diverse populations	○	○	○	○	○
B5.4 Convey information to professionals, personnel and the public using a variety of approaches (e.g. reports, presentations, press releases, emails, social media, etc.)	○	○	○	○	○
B5.5 Communicate effectively in writing and orally, in person and through electronic means, with linguistic and cultural proficiency	○	○	○	○	○
B5.6 Maintain relationships with diverse community partners to assist with communicating preparedness planning and population-specific messages	○	○	○	○	○
B5.7 Verify the credibility of information and sources.	○	○	○	○	○

[Part B] Knowledge, Skills and Abilities required for specific PHEOC roles and functions

B6. Logistics function

B6.0 Is your job mandate partly involved with PHEOC's *Logistics* function?

◯ Yes ◯ No ◯ Not sure

Yes / Not sure ⟶ Jump to B6.1

No ⟶ Skip to B7.0

[Part B] Knowledge, Skills and Abilities required for specific PHEOC roles and functions

Please note that we are collecting these information solely to analyze the strength and gaps in your PHEOC's implementation, so as to document the best practices and reflect future improvement for your capacity building programs. The result of this survey will NOT be shared with your PHEOC supervisors.

In this regard, please answer the questions based on your honest situation. Please do NOT select randomly or select 5 for all the self-rated questions.

B6. Logistics function

*** In general, how well do you think you can fulfill the following subjects?**
(1 = very poorly, 5 = very well)

	1	2	3	4	5
B6.1 Support information systems development and perform IT systems operations and maintenance	○	○	○	○	○
B6.2 Administer procurement procedures and protocols, particularly those most relevant to public health	○	○	○	○	○
B6.3 Plan and implement distribution systems and use inventory management systems	○	○	○	○	○
B6.4 Know hazardous materials regulations	○	○	○	○	○
B6.5 Practice supply chain management	○	○	○	○	○
B6.6 Know human resource policy, procedures, recruitment and rostering practices	○	○	○	○	○
B6.7 Provide or administer facilities maintenance services	○	○	○	○	○
B6.8 Develop and maintain a database of logistics, etc	○	○	○	○	○
B6.9 Oversee provision of all emergency response facilities, supplies, services and resources	○	○	○	○	○
B6.10 Ensure that response personnel have sufficient food and potable water	○	○	○	○	○
B6.11 Order, receive, store and distribute supplies and equipment, and coordinate procurement contracts with the finance section	○	○	○	○	○
B6.12 Prepare and maintain logistics management plans and SOPs	○	○	○	○	○
B6.13 Provide services to support emergency operations	○	○	○	○	○

[Part B] Knowledge, Skills and Abilities required for specific PHEOC roles and functions

B7. Finance and Administration function

B7.0 Is your job mandate partly involved with PHEOC's *Finance and Administration* function?

◯ Yes ◯ No ◯ Not sure

Yes / Not sure ⟶ Jump to B7.1

No ⟶ Skip to Part C

[Part B] Knowledge, Skills and Abilities required for specific PHEOC roles and functions

Please note that we are collecting these information solely to analyze the strength and gaps in your PHEOC's implementation, so as to document the best practices and reflect future improvement for your capacity building programs. The result of this survey will NOT be shared with your PHEOC supervisors.

In this regard, please answer the questions based on your honest situation. Please do NOT select randomly or select 5 for all the self-rated questions.

B7. Finance and Administration function

* **In general, how well do you think you can fulfill the following subjects?**
(1 = very poorly, 5 = very well)

	1	2	3	4	5
B7.1 Utilize records management systems that satisfy agency standards for important documents and financial records	○	○	○	○	○
B7.2 Distinguish between different types of electronic information and sources	○	○	○	○	○
B7.3 Describe and utilize the financial planning, budgetary and cash flow processes of the agency	○	○	○	○	○
B7.4 Design and implement financial plans for assigned operational projects	○	○	○	○	○
B7.5 Prepare proposals for funding (e.g. to foundations, government agencies, corporations, etc.)	○	○	○	○	○
B7.6 Negotiate contracts and other agreements for programmes and services	○	○	○	○	○
B7.7 Process compensation claims (incentives, insurance, expenses	○	○	○	○	○

This concludes Part B of the Individual Capacity Survey.
Please proceed to next page to complete the last part.

[Part C] Core competencies for public health emergency management

Thank you for completing Part B of the survey.

In Part C, you will be asked to review the core competencies defined by the WHO PHEOC training guidance.

Several statements will be listed below.
In each statement, you will be provided with a scale from 1-5,
1 = "the statement doesn’t fit me at all",
5 = "the statement fits me perfectly".
Select 0 if the statement is not applicable to your job mandate.

Please select how much you think the statements fit your capacity.

[Part C] Core competencies for public health emergency management

Please note that we are collecting these information solely to analyze the strength and gaps in your PHEOC's implementation, so as to document the best practices and reflect future improvement for your capacity building programs. The result of this survey will NOT be shared with your PHEOC supervisors.

In this regard, please answer the questions based on your honest situation. Please do NOT select randomly or select 5 for all the self-rated questions.

C1. Leadership

*** C1. From scale 1-5, how much do you believe the following statements fit your *Leadership* capacity?**

	1	2	3	4	5	0
C1.1 I can develop strategic plans to define the organization's vision, mission, values, structure, goals, objectives, performance measures, outcomes, resources, budget, and continuous quality improvement methods to align with the people and processes and to build capacity.	○	○	○	○	○	○
C1.2 In activity designing, I can well align the activities with the organization's vision, mission, values, goals, and strategies.	○	○	○	○	○	○
C1.3 I could apply knowledge of change management principles to impact organizational development and improvement.	○	○	○	○	○	○
C1.4 I am able to assemble interdisciplinary teams to collaboratively generate solutions.	○	○	○	○	○	○
C1.5 I always try to apply leadership methods when interacting with partners, teams and staff.	○	○	○	○	○	○
C1.6 I always apply self-control and composure to manage relationships constructively and with professionalism while under pressure during a crisis.	○	○	○	○	○	○
C1.7 I would apply decision making and problem-solving methods to develop solutions to problems and to adjust systems accordingly.	○	○	○	○	○	○

[Part C] Core competencies for public health emergency management

Please note that we are collecting these information solely to analyze the strength and gaps in your PHEOC's implementation, so as to document the best practices and reflect future improvement for your capacity building programs. The result of this survey will NOT be shared with your PHEOC supervisors.

In this regard, please answer the questions based on your honest situation. Please do NOT select randomly or select 5 for all the self-rated questions.

C2. Overall Emergency Management Framework

* **C2. From scale 1-5, how much do you believe the following statements fit your capacity in *understanding and applying overall emergency management framework*?**

	1	2	3	4	5	0
C2.1<br>I have comprehensive knowledge of public health emergency management doctrines, e.g. the WHO Framework for PHEOCs, the International Health Regulations, the national preparedness frameworks, and the national incident management systems of my country.	○	○	○	○	○	○
C2.2<br>I feel confident in applying the knowledge of public health and emergency management authorities, including laws, regulations, guidelines, treaties, and other policy documents, and act within the scope of those authorities.	○	○	○	○	○	○

[Part C] Core competencies for public health emergency management

Please note that we are collecting these information solely to analyze the strength and gaps in your PHEOC's implementation, so as to document the best practices and reflect future improvement for your capacity building programs. The result of this survey will NOT be shared with your PHEOC supervisors.

In this regard, please answer the questions based on your honest situation. Please do NOT select randomly or select 5 for all the self-rated questions.

C3. Emergency Management

*** C3. From scale 1-5, how much do you believe the following statements fit your capacity in *Emergency management*?**

	1	2	3	4	5	0
C3.1<br>I am familiar with conducting threat and hazard identification and risk assessment.	○	○	○	○	○	○
C3.2<br>I can develop strategic and operational plans using key planning principles.	○	○	○	○	○	○
C3.3<br>I am equipped with knowledge and skills to assess and coordinate emergency management operations to maintain an in-depth comprehension of the operational environment to anticipate, identify and report public health threats.	○	○	○	○	○	○
C3.4<br>I am confident in integrating foundational emergency management principles into the national public health systems structure.	○	○	○	○	○	○
C3.5<br>I know how to synthesize and share information and data to inform decision making across public health emergency management organizational functions.	○	○	○	○	○	○
C3.6<br>I can properly conduct logistics and resource management activities.	○	○	○	○	○	○
C3.7<br>I am well equipped with knowledge of public health emergency management technologies and systems.	○	○	○	○	○	○

[Part C] Core competencies for public health emergency management

Please note that we are collecting these information solely to analyze the strength and gaps in your PHEOC's implementation, so as to document the best practices and reflect future improvement for your capacity building programs. The result of this survey will NOT be shared with your PHEOC supervisors.

In this regard, please answer the questions based on your honest situation. Please do NOT select randomly or select 5 for all the self-rated questions.

C4. Emergency Management Communication

* **C4. From scale 1-5, how much do you believe the following statements fit your capacity in *Emergency management communication*?**

	1	2	3	4	5	0
C4.1 I can integrate crisis and emergency risk communication principles into all stages of an incident.	○	○	○	○	○	○
C4.2 I feel confident managing and sharing information across internal public health partners properly.	○	○	○	○	○	○
C4.3 I feel confident managing and sharing information across external public health partners properly.	○	○	○	○	○	○
C4.4 I am well aware of how to apply comprehensive knowledge of emergency alert systems to ensure crisis information and guidance are delivered to partners, stakeholders and the public.	○	○	○	○	○	○
C4.5 I know clearly how to applies knowledge of interoperable and integrated communication strategies to facilitate information sharing during an emergency.	○	○	○	○	○	○

[Part C] Core competencies for public health emergency management

Please note that we are collecting these information solely to analyze the strength and gaps in your PHEOC's implementation, so as to document the best practices and reflect future improvement for your capacity building programs. The result of this survey will NOT be shared with your PHEOC supervisors.

In this regard, please answer the questions based on your honest situation. Please do NOT select randomly or select 5 for all the self-rated questions.

C5. Partnership and Collaboration

* **C5. From scale 1-5, how much do you believe the following statements fit your capacity in *Partnership and Collaboration*?**

	1	2	3	4	5	0
C5.1 I feel confident in collaborating with governmental and non-governmental organizations to ensure coordination of preparedness and response activities.	○	○	○	○	○	○
C5.2 I can properly develop and maintain relationships within internal teams and external partners.	○	○	○	○	○	○
C5.3 I always collaborate with individuals, teams and partners to obtain feedback, solve problems and handle challenges within an emergency management environment.	○	○	○	○	○	○
C5.4 I would always try to apply knowledge of partner organizations' capabilities, mission, roles, responsibilities and priorities when developing public health emergency standards, guidelines and protocols.	○	○	○	○	○	○

[Part C] Core competencies for public health emergency management

Please note that we are collecting these information solely to analyze the strength and gaps in your PHEOC's implementation, so as to document the best practices and reflect future improvement for your capacity building programs. The result of this survey will NOT be shared with your PHEOC supervisors.

In this regard, please answer the questions based on your honest situation. Please do NOT select randomly or select 5 for all the self-rated questions.

C6. Training Development and Facilitation

* **C6. From scale 1-5, how much do you believe the following statements fit your capacity in *Training Development and Facilitation*?**

	1	2	3	4	5	0
C6.1 I can apply systematic methods to analyze training needs, and to design and develop educational briefings, presentations and materials.	○	○	○	○	○	○
C6.2 I am able to develop instructional strategies and methods to use for achieving training goals and objectives, including examples and practice activities.	○	○	○	○	○	○
C6.3 I feel confident in delivering emergency management training according to instructional strategies.	○	○	○	○	○	○

[Part C] Core competencies for public health emergency management

Please note that we are collecting these information solely to analyze the strength and gaps in your PHEOC's implementation, so as to document the best practices and reflect future improvement for your capacity building programs. The result of this survey will NOT be shared with your PHEOC supervisors.

In this regard, please answer the questions based on your honest situation. Please do NOT select randomly or select 5 for all the self-rated questions.

C7. Evaluation

* **C7. From scale 1-5, how much do you believe the following statements fit your capacity in *Evaluation*?**

	1	2	3	4	5	0
C7.1<br>I know well how to develop tools, metrics and methods for evaluating progress toward achieving preparedness and response objectives.	○	○	○	○	○	○
C7.2<br>I can conduct evaluation on capacities and provide recommendations to address identified gaps.	○	○	○	○	○	○
C7.3<br>I fully understand how to evaluate processes and procedures to assess preparedness and response actions.	○	○	○	○	○	○
C7.4<br>I am confident to apply exercise program management principles to validate and improve operational capability.	○	○	○	○	○	○
C7.5<br>I can apply evaluation methods to assess levels of learning that occur as a result of training presentations, briefings, and materials.	○	○	○	○	○	○
C7.6<br>I know clearly how to develop strategies and tools to ensure sustainability of the Emergency Management program.	○	○	○	○	○	○

Congratulations!

You have completed all questions for the Individual Capacity Survey.
Please click "Submit" to save your answers.

Thank you very much for your participation.
Have a nice day!

If you have any other comments about the questions in this survey, please leave below:
