# Supplementary material for: Implementing public health emergency operations centres according to an international framework in Ethiopia, Nigeria, and Senegal: Best practices and achievements, 2021
Source: PLOS Glob Public Health. 2026 Apr 27;6(4):e0006211. doi: 10.1371/journal.pgph.0006211 (PMC13119900; doi:10.1371/journal.pgph.0006211)
Supplement: Attachment — Submitted filename: Rebuttal letter_Responses to editor and reviewers.docx [file pgph.0006211.s004.docx]

**Rebuttal letter – Responses to comments from the editor and reviewers**

Many thanks for your constructive feedback. Below, we show how we tried to address all of the concerns from the editor and reviewers in order to improve our paper.

Journal Requirements:

1. Please include a complete copy of PLOS’ questionnaire on inclusivity in global research in your revised manuscript. Our policy for research in this area aims to improve transparency in the reporting of research performed outside of researchers’ own country or community. The policy applies to researchers who have travelled to a different country to conduct research, research with Indigenous populations or their lands, and research on cultural artefacts. The questionnaire can also be requested at the journal’s discretion for any other submissions, even if these conditions are not met.  Please find more information on the policy and a link to download a blank copy of the questionnaire here: https://journals.plos.org/globalpublichealth/s/best-practices-in-research-reporting. Please upload a completed version of your questionnaire as Supporting Information when you resubmit your manuscript.

We have completed the inclusivity in global research questionnaire and uploaded to the system as supporting information.

2. You indicated that ethical approval was not necessary for your study. We understand that the framework for ethical oversight requirements for studies of this type may differ depending on the setting and we would appreciate some further clarification regarding your research. Could you please provide further details on why your study is exempt from the need for approval and confirmation from your institutional review board or research ethics committee (e.g., in the form of a letter or email correspondence) that ethics review was not necessary for this study? Please include a copy of the correspondence as an "Other" file."

Check this with SENAIT on how to put the statement for this request.

3. Please provide a complete Data Availability Statement in the submission form, ensuring you include all necessary access information or a reason for why you are unable to make your data freely accessible. If your research concerns only data provided within your submission, please write "All data are in the manuscript and/or supporting information files" as your Data Availability Statement.

Raw data will be uploaded to the system recommended by the journal.

4. We have amended your Competing Interest statement to comply with journal style. We kindly ask that you double check the statement and let us know if anything is incorrect.

In agreement with the amendment.

5. Please review your reference list to ensure that it is complete and correct. If you have cited papers that have been retracted, please include the rationale for doing so in the manuscript text, or remove these references and replace them with relevant current references. Any changes to the reference list should be mentioned in the rebuttal letter that accompanies your revised manuscript. If you need to cite a retracted article, indicate the article’s retracted status in the References list and also include a citation and full reference for the retraction notice.
We reviewed the reference list and we believe we have included all the references used in the manuscript.
Additional Editor Comments (if provided):

Please review the detailed comments from each reviewer, and address all points in your response. Please pay particular attention to the comments pertaining to the methods, to ensure clarity and transparency. Both reviewers have also highlighted the importance of aligning the findings with requirements under the International Health Regulations, and particular the Joint External Evaluation process, so please ensure these suggestions are integrated into the revised paper.

Yes, we agree. We have tried to address the reviewers’ comments point by point including the major comments given around method.

Reviewer #1:

Background
It is important to document Nigeria, Senegal and Ethiopia (especially Nigeria because its in West Africa and reported EVD cases) emergency preparedness and response system during EVD outbreak in West Africa. This will set the tone of how far these countries have come in strengthening their PHEOC before COVI-19 and other public health emergencies (Lassa fever polio, EVD). A Nigeria Polio PHEOC paper could shade some light on PHEOC in Nigeria before COVID-19 https://pubmed.ncbi.nlm.nih.gov/34711580/
Information on JEE recommendations for 3 countries will be useful for your background to justify choice of three countries beyond their interest to share experiences.
Methods
It would be informative if survey tools are provided as supporting information
Individual level data- A total of 76 PHEM staff were invited to complete the survey: break done the 76 PHEM staff by countries to show representation.

It now added the number and proportion of staff who were interviewed by country and now put as … A total of 76 staff (Ethiopia: 36, Nigeria, 15 and Senegal: 22) were invited to complete the individual survey, which included 124 questions..

For part C of the questionnaire, it will be informative to list core competencies required of response personnel.

We agree with the suggestion and the sentence is now rephrased to reflect the suggestion … Part C addressed the core competencies required of response personnel to ensure effective management of PHEs including leadership capacity, capacity in understanding emergency management principles and doctrines, integrating crisis and emergency risk communication to an incident, training facilitation and evaluation.
Results
A table on characteristics of respondents by countries will be informative
Institutional level capacities:
Legal instruments, Plans and procedures
It’s not clear whether countries have multi-hazard plans, incident or hazard-specific response and management plans.

It now added in lines 159-161 to reflect existence of plans in these countries … rephrased the sentence as … Plans and procedures including PHEOC operations and management handbooks, the multi-hazard, and hazard-specific preparedness and response plans helped to ensure clear guidance, coordination and communication among the various actors.
Human resources, training, and exercise
Since you reported that all 3 countries have a database of staff trained on PHEM, it will be informative to document total core and surge staff trained on various PHEM trainings per country. This information can be added to Table 1. To Table 1, you may also add more variables like duration of the training, and type of training (virtual, online or in-person).
“The Nigeria PHEOC implemented a flexible plan that allowed a group of resilient employees to work in multiple technical areas at the same time” does this mean the employees were doing other technical work outside PHEOC in addition to their PHEOC roles or they work in multiple technical areas within PHEOC. Please clarify.

We wanted to say the response personnel were working in multiple technical areas within the PHEOC operations and we now rephrased it as … The Nigeria PHEOC implemented a flexible plan that allowed response personnel to work in multiple technical areas at the same time within the PHEOC and implementing an updated surge staff recruitment plan helped to address the gap.
Data and information
“Compared with the other two countries, Ethiopia still needs to improve its data processing and analysis capabilities”- This is more of a recommendation but in results of the paper. Since this is results section, point out the Ethiopia gaps in data processing and analysis capabilities.

Technology and physical infrastructure
Besides, computers, phones, TV plasma screens, and radio systems, already listed, the internet connectivity is important technology infrastructure to improve communication, it is important you add it to the list if it was available.

Individual-level capacities:
A total of 76 PHEM staff were invited to complete the survey: What was the response rate?
“Knowledge and concepts of PHEOC operations”- it’s important to note that this is self- reported. It should read “Self-reported knowledge and concepts of PHEOC operations”, change SI Table title to read the same

We have changed in the manuscript and read now as self-reported knowledge and concepts of PHEOC operations and same in the S1 Table.
“PHEOC function-specific roles” to read “Self-reported PHEOC function-specific roles”

We have changed in the manuscript and read now as self-reported PHEOC function-specific roles.
“Readiness and Response to COVID-19” to read self-reported readiness and response to COVID-19

We have changed in the manuscript and read now as self-reported readiness and response to COVID-19.

Discussion
“The results showed that PHEOC plays a critical role in preparing for and responding to public health emergencies”- It will be informative to explore some enablers that made PHEOC worked for COVID-19. This could be other public health events for which PHEOC was activated to coordinate response before COVID-19 pandemic

Reviewer #2: Implementing public health emergency operations centres according to an international framework in Ethiopia, Nigeria, and Senegal: Best practices and achievements, 2021.

The paper meets the publication criteria by addressing a relevant public health issue and contributing valuable insights into PHEOC implementation in multiple countries.
The paper appears technically sound, and the data provided support the conclusions drawn about the implementation and effectiveness of PHEOCs in Ethiopia, Nigeria, and Senegal.
The paper also describes methodologically rigorous research with appropriately drawn conclusions based on the comparative analysis of PHEOCs in three countries.
The paper is clear, well-organized, and written in standard English, but the punctuation in the final outputs should be critically examined after addressing the comments.
The paper did not mention data availability beyond the analyzed data, so confirming availability and access to the data will be good.
We have updated the availability of data and materials sub-section in the manuscript to reflect data availability and uploaded in line with journal’s data platform .
Abstract
ROW 48: Consistency in writing out COVID-19 correctly with the hyphen
It is now written in full and consistent throughout the manuscript with a hyphen.
Main Paper
ROW 90: Nigeria is missing at the beginning of the name of NCDC

Yes, it is now added Nigeria before the Centres …in the manuscript.
ROW 123: This is the first time PHEM will be referenced and should be written in full.

Yes, it is now put in full as it’s the first time it appears … public health emergency management (PHEM)
PHEM staff were not included in the cadre of staff under data collection, so please update as applicable. Also, under the background section, a sentence on the concept of PHEM implementation will serve as additional insight for a reader to know that these countries have staff dedicated as PHEM.
ROW 129: Write PHE in full since this is the first time before the abbreviation.

We addressed this comment and rephrased the last words and read as …management of PHEs.
ROW 136: Include a hyphen or space on “selfrating”

In the manuscript, hyphen is included to separate the two words.
ROW 152: Is this task force for just Nigeria or both countries, as it is reflected as singular?

The task force refers to all countries and now made some edits to reflect it is for the countries.
ROW 160: Closed the bracket with the abbreviation of RRTs.

Yes, we now add a bracket to close it … (RRT) in the manuscript.
ROW 161: The statement is unclear. Do you mean “permission to respond to personnel who support response operations”? Or “permission to response personnel who support response operations”?

We mean permission to response personnel who support response operations and we have added who to make the sentence clear as suggested.
ROW 166: Access is one thing, was the writer able to elicit information about utilization to reflect the number of people who leveraged these opportunities to build their capacities and the exercises? If yes, kindly reflect that.

We agree with the recommendation to consider the take-up of trainings. However, we were concerned with the availability of training opportunities offered at various levels to enhance the competencies of the IMS workforce. We know from experience that training opportunities helped to develop abilities, but we did not take into account their impact.

ROW 184: The statement speaks to using SORMAS for managing suspected cases. Any information on how the confirmed cases interface with SORMAS data for PHEOC use since confirmed cases will be coming from the laboratory?
ROW 187: Does this also support laboratory information to complete the loop from suspected cases to confirm cases needed by the PHEOCs to act? Please reflect this if the information is available.
ROW 209: “Concept” should be plural.

Yes, it is now expressed in plural in the manuscript.
ROW 25I: Add a hyphen before 19 in COVID-19

There is a hyphen between the two words in the manuscript.
ROW 263: “Establish” should be in the past tense in the sentence all three countries establish their……Yes, it is now expressed the statement in past sentence form.
ROW 269: This is a bit unclear. Were the meetings for the PHEOC or the Presidential Task Force, or were they both combined? If different, insert a sentence on how they operated synergistically.
ROW 271: “Holding” in the sentence “The PHEOC in Senegal was holding daily meetings….” should be in the past tense/

Yes, it is now in past sentence and put as…The PHEOC in Senegal held daily meetings to discuss….
ROW 307: Correct to reflect Nigeria and not Niger in the sentence “It was found that Ethiopia had a standing policy group however, Niger and Senegal had ad…”

Yes it is corrected now, it is Nigeria not Niger.
ROW 361: “Skill and Attitude” in the sentence “knowledge, skill, and attitude should be plural.

It is corrected now, we changed it to plural form, … knowledge, skills and attitudes …
ROW 369: This is the first and only time that JEE is being thrown in. Can you write it in full? In addition, is there a way to reflect implementing PHEOC in line with JEE recommendations in either the discussion or result sections, too, so it is not just hanging loosely here?

In align with the suggestion to have a link between the PHEOC and JEE, we added sentences in the background to provide clarifications. Furthermore, the Joint External Evaluation (JEE) conducted in the three countries indicated critical gaps in establishing effective coordination for the management of PHEs.
